# Stage-Specific Associations Between Respiratory Pathogens and Mortality in Sheep: A Multivariate Analysis

**DOI:** 10.3390/ani16193028

**Published:** 2026-09-26

**Authors:** Antonio Romero-Salmoral, Manuela Hernández-García, Francisco Javier Navas González, Carmen Tarradas Iglesias, Belen Huerta Lorenzo, Inmaculada Luque Moreno, Eva Boyer-Bustamante, Francisco Jurado-Martos

**Affiliations:** 1 Animal Health Research Group: Diagnosis and Control of Diseases (SANDYC), Unidad de Investigación Competitiva (UIC) Zoonosis y Enfermedades Emergentes ENZOEM, Department of Animal Health, International Excellence Agrifood Campus ‘CeiA3’, University of Córdoba, 14071 Córdoba, Córdoba, Spain; antromsal98@gmail.com (A.R.-S.); sa1taigc@uco.es (C.T.I.); sa2hulob@uco.es (B.H.L.); sa1lumoi@uco.es (I.L.M.); 2 Department of I+D+I, Centro Tecnológico de Investigación Agroalimentaria (CICAP), 14400 Pozoblanco, Córdoba, Spain; mhernandez@cicap.es (M.H.-G.); fjurado@cicap.es (F.J.-M.); 3 Department of Genetics, International Excellence Agrifood Campus ‘CeiA3’, University of Córdoba, 14071 Córdoba, Córdoba, Spain; 4 International Doctoral School in Agri-Food (eidA3), Rabanales University Campus, University of Cordoba, 14071 Córdoba, Córdoba, Spain; ebobuvet@gmail.com

**Keywords:** bronchoalveolar lavage, real-time PCR, *Mannheimia haemolytica*, *Mesomycoplasma ovipneumoniae*, *Pasteurella multocida*

## Abstract

Ovine Respiratory Complex (ORC) is a major cause of lamb mortality in intensive and semi-intensive sheep production systems. Its multifactorial etiology, involving bacterial and viral pathogens together with host and environmental factors, complicates disease control and limits the effectiveness of conventional preventive measures. Although respiratory pathogens are commonly detected in healthy lambs, their contribution to mortality at different production stages remains poorly understood. This study evaluated the relationships between respiratory pathogens and lamb mortality using canonical multivariate analysis. A total of 200 bronchoalveolar lavage samples were collected from apparently healthy lambs aged 50–60 days during winter (n = 80) and summer (n = 120) on 10 commercial sheep farms in southwestern Spain. Samples were analyzed by real-time PCR for the detection of major bacterial and viral respiratory pathogens, and mortality records from farms and feedlots during 2022–2023 were incorporated into the analysis. A high herd-level prevalence was detected for *Pasteurella multocida*, *Mannheimia haemolytica*, *Bibersteinia trehalosi*, and *Mesomycoplasma ovipneumoniae*, whereas *Histophilus somni*, Orf virus, and parainfluenza virus type 3 showed intermediate prevalence. No significant seasonal differences were observed in pathogen prevalence. ORC-associated pathogens accounted for approximately 20% of the variance in mortality, suggesting that non-infectious factors also play an important role in lamb losses. Canonical analysis identified stage-specific multivariate patterns, with *M. ovipneumoniae* and *P. multocida* contributing most strongly to the canonical dimension characterized by on-farm mortality, whereas *M. haemolytica*, together with *P. multocida* and parainfluenza virus type 3 (PIV3), contributed most strongly to the dimension characterized by feedlot mortality.These findings improve our understanding of the epidemiology of ORC and support the development of targeted surveillance, prevention, effective vaccination of ewes against the serotypes circulating within farms, and management strategies to reduce lamb mortality, improve animal welfare, and enhance the sustainability of sheep production.

## Simple Summary

Ovine respiratory disease is one of the main causes of lamb mortality and reduces animal welfare and farm profitability. However, it remains difficult to identify which microorganisms are most strongly associated with deaths at different production stages because several pathogens are commonly found in healthy animals. Here, we analyzed lung wash samples from 200 apparently healthy lambs from commercial sheep farms in Spain and compared the presence of the main respiratory pathogens with mortality rates recorded on farms and in feedlots using a multivariate statistical approach. Our analyses showed that *Mesomycoplasma ovipneumoniae* and *Pasteurella multocida* were mainly associated with on-farm mortality, whereas *Mannheimia haemolytica* was the principal pathogen associated with feedlot mortality. These findings demonstrate that the importance of respiratory pathogens changes throughout the production cycle and highlight the need for stage-specific health monitoring and effective vaccination of ewes against the isolates circulating within farms, and management strategies to reduce lamb mortality, improve animal welfare, and enhance the sustainability of sheep production.

## 1. Introduction

Sheep meat production is a key economic activity in the southwest of the Iberian Peninsula and is commonly conducted under extensive or semi-intensive systems, contributing to the stability of rural economies and environmental sustainability [1,2]. The fattening period may take place either on the same premises where lambs are born or in separate feedlots [3]. During this stage, animals are susceptible to various diseases that can substantially compromise their health and welfare. Among these, respiratory diseases are a major concern, causing significant economic losses through high morbidity and mortality, reduced weight gain, and lung lesions that may result in carcass condemnation at slaughter [3,4,5,6].

The Ovine Respiratory Complex (ORC) is a multifactorial syndrome arising from complex interactions among infectious agents, the host, and environmental and management factors, which collectively complicate effective prevention and control [7,8]. ORC is particularly important during the fattening period, when morbidity and mortality rates of up to 75% and 50%, respectively, have been reported [3]. Clinical disease commonly manifests as acute pneumonia, which may progress to systemic infection and death [9,10].

A range of stressors, including dietary changes following weaning, transport to feedlots, regrouping of lambs from different origins, overcrowding, and adverse environmental conditions (e.g., inadequate ventilation, extreme weather, and limited access to feed or water), may exacerbate ORC, particularly in the presence of co-infections [6,11]. Studies have also documented a marked increase in nasopharyngeal microbiota diversity during the first week following feedlot arrival [3,12].

Members of the family Pasteurellaceae, particularly *Pasteurella multocida*, *Mannheimia haemolytica*, and *Bibersteinia trehalosi*, are consistently implicated as major bacterial pathogens associated with ORC. These bacteria commonly occur as commensals in the upper respiratory tract but may become pathogenic under conditions of stress or immunosuppression [7,13,14]. Their considerable serotype diversity may have important clinical and immunological implications [11]. *Mesomycoplasma ovipneumoniae* has also been identified as a primary pathogen that may predispose animals to secondary infections involving Pasteurellaceae [15,16,17]. Other microorganisms, such as *Histophilus somni*, which is more commonly associated with bovine respiratory disease, are increasingly being detected in sheep and may contribute to respiratory disease [10,18,19]. Viral agents, including PIV3 and Orf virus, may further exacerbate disease severity by damaging respiratory tissues and/or impairing host immune responses [19,20,21].

Understanding the relationships between these pathogens and lamb mortality across different production stages is essential for improving our understanding of ORC epidemiology and its clinical relevance [5]. Canonical correlation analysis (CCA) is a multivariate statistical approach that enables the simultaneous investigation of relationships between two sets of biological variables and has been successfully applied in related studies [22]. By identifying linear combinations of variables within each set that maximize their mutual correlation, CCA provides an integrative framework for investigating complex biological relationships that may not be adequately captured through separate univariate analyses. Regularized extensions of CCA (RCCA) further enhance this approach when variables within either set are highly correlated, as regularization can reduce the effects of multicollinearity and improve the stability of the estimated multivariate relationships.

Although previous research has described the prevalence and pathogenic roles of individual ORC-associated microorganisms, few studies have quantitatively evaluated their combined association with mortality across different production stages. In particular, the relationship between pathogen detection in apparently healthy lambs and subsequent stage-specific mortality remains poorly understood. A multivariate approach may therefore help disentangle the complex relationships between pathogen occurrence and mortality.

Therefore, the aim of this study was to quantify and model the multivariate relationships between ORC-associated pathogens and mortality across different production stages in livestock and feedlot systems, with the objective of identifying stage-specific patterns associated with mortality risk.

## 2. Materials and Methods

### 2.1. Study Design and Sample Collection

This study was conducted in a region located in the south-western of Spain, with a continental mediterranean climate, characterized by hot and dry summers, with temperatures higher to 40 °C, cold winters with frequent frosts, and concentrated rainfall around winter and autumn (500–600 mm annually) [23]. Meat sheep are usually raised in the Dehesa, a sustainable and environmentally friendly system of production. The animals are reared in semi-intensive conditions. Generally, after weaning the animals are transported to the feedlot to be fattened during a period of 21 days and subsequent slaughter in a local slaughterhouse.

A total of ten sheep meat production farms were selected by their participation in the Immunity UP: improvement of the health status of extensive sheep project. The selection criteria included mortality records and accessibility for sampling.

Between February and September 2023, a total of 200 bronchoalveolar lavage (BAL) samples were collected from apparently healthy lambs aged 50–60 days. Sampling was conducted in two phases: BAL samples from eight lambs per farm (n = 80 samples) were obtained in winter (between February and April) and twelve samples per farm in summer (between June and September) (n = 120 samples). A larger sample size was collected during summer due to the expected higher prevalence in this season [24]. Lambs were nasally intubated using a sterile feeding tube (2.7 mm in diameter and 50 cm in length). A total of 20 mL of sterile physiological saline solution was instilled via a sterile syringe with a Luer-Lock system, and the fluid was immediately aspirated. The recovery of cellular and non-cellular components throughout the lung parenchyma was achieved [25]. The recovered samples were transferred to Falcon™ (Corning Inc., Corning, NY, USA) sterile tubes and stored under refrigeration (4 ± 1 °C) and sent to the laboratory on the same day for further analysis.

### 2.2. Mortality Data Collection

Mortality data were obtained from farm or feedlot records (proportion of dead lambs with respiratory symptoms versus total lambs born or delivered, respectively) for the years 2022 and 2023. The overall mortality rate per herd was determined by calculating the average of the annual mortality rates during this period.

### 2.3. Microbiological Analysis of BAL

BAL samples were processed within 24 h of collection using the KingFisher™ mL automated nucleic acid purification system (Life Technologies, Darmstadt, Germany) in combination with the MagMAX™ CORE extraction kit (Life Technologies, Darmstadt, Germany), following the manufacturer’s recommendations. The extracted nucleic acids were stored at −80 °C until further analysis.

Each sample was analysed by real-time PCR using commercially available kits for the detection of *M. haemolytica*, *P. multocida*, *B. trehalosi*, *M. ovipneumoniae*, *H. somni*, Orf virus (Genetic PCR Solutions^®^ (Orihuela, Spain)), and PIV3 (Exopol, Zaragoza, Spain). Reactions were performed on a QuantStudio 5^®^ real-time thermal cycler (Applied Biosystems^®^, Waltham, MA, USA) in a final volume of 20 μL, comprising 5 μL of extracted nucleic acids and 15 μL of master mix. Positive and negative controls, including negative extraction controls (extraction blanks), a no-template control (NTC), and the assay internal control supplied with the commercial kits were included in each real-time PCR run according to the manufacturers’ instructions. The internal control was used to verify correct assay performance and to detect potential PCR inhibition; it was not used for normalization of quantification cycle (Cq) values between samples. Cq values were not normalized to a host reference gene or to the volume of BAL fluid recovered from each animal. Cq values were recorded for each sample, with a positivity threshold set at Cq ≤ 38. Samples without detectable amplification were assigned a Cq value of 40 for statistical analysis. Raw Cq values were entered directly into the statistical analyses without inversion or other mathematical transformation; therefore, higher Cq values represent lower measured molecular signals. No standards curves or calibrators of known target concentration were used; therefore, Cq values were not interpreted as absolute pathogen concentration.

Pathogen prevalence was calculated as the proportion of positive samples among the total number of samples tested at the individual and seasonal levels (Table 1). Exact 95% confidence intervals (CIs) for prevalence estimates were calculated using the Clopper–Pearson exact binomial method, based on the number of positive samples and the corresponding sample size. Herd-level prevalence was expressed as the proportion of herds in which the pathogen was detected among the 10 herds studied.

### 2.4. Statistical Analysis

An analytical cross-sectional study was carried out to investigate the relationship among the ovine mortality and the presence of different pathogens related to the ovine respiratory complex. The sample size was determined using the software Winepi 2.0 (Veterinary Faculty of Veterinary, University of Zaragoza, 2006, Spain), considering a confidence level of 95%, a margin of error of 7%, and an expected prevalence of 50% (http://www.winepi.net/ (accessed on 2 July 2026)).

#### 2.4.1. A Priori Assumptions

Regularized canonical correlation analysis (RCCA) was used to assess linear relationships between the two sets of variables and requires consideration of distributional assumptions, variance homogeneity, sample size, and multicollinearity [26]. Normality and homoscedasticity were assessed using the Shapiro-Wilk (for samples up to n = 5000)/D’Agostino-Pearson tests [27,28] and Levene’s test [29], respectively; because these assumptions were not consistently satisfied, permutation-based inference was applied to assess the significance of the canonical correlations [30]. Potential outliers were evaluated using the Robust regression and Outlier Removal (ROUT) method with a false discovery rate threshold of 1% [31], while multicollinearity was assessed using the variance inflation factor (VIF), with a threshold of 5 [32]. The available sample size was considered adequate relative to the number of variables included in the analysis, consistent with the recommended minimum of approximately 10 observations per variable [33].

#### 2.4.2. Assessment of the Within-Herd Hierarchical Structure of Pathogen Data

Because pathogen Cq values were measured at the individual-lamb level, whereas mortality was recorded at the herd level, the potential clustering of pathogen observations within herds was assessed before interpretation of the multivariate analyses. The dataset comprised 200 lambs distributed across 10 herds, with 20 lambs per herd. Two herd-level mortality variables were available: mortality rate during the farm stage and mortality rate during the feedlot stage. Thus, all lambs belonging to the same herd shared the same values for the corresponding mortality variables.

For pathogen detection analyses, a Cq value <40 was considered positive, whereas a Cq value of 40 was considered non-detected. Individual-level prevalence was calculated as the proportion of positive lambs among the 200 animals, whereas herd-level prevalence was calculated as the proportion of herds containing at least one positive animal. These measures were reported separately to describe the distribution of pathogen detection at the individual and herd levels.

The degree of within-herd clustering of pathogen detection was quantified using the intraclass correlation coefficient (ICC), with herd included as the clustering factor. For each microorganism, the ICC was calculated as the proportion of the total variance attributable to differences between herds:

$$ICC=\frac{\sigma_{between}^{2}}{\sigma_{between}^{2}+\sigma_{within}^{2}}$$

where $\sigma_{between}^{2}$ represents the between-herd variance and $\sigma_{within}^{2}$ represents the within-herd variance. ICC values were interpreted quantitatively as estimates of the proportion of variability attributable to herd-level differences rather than according to universal reliability thresholds, because the purpose of the analysis was to characterize clustering rather than measurement reliability.

To quantify the potential effect of within-herd clustering on the nominal number of observations, the design effect was calculated as:

$$DE=1+\left(m-1\right)\times ICC$$

where m=20 represents the number of lambs per herd. The corresponding effective sample size was estimated as:

$$n_{eff}=\frac{n}{DE}$$

where n=200 represents the total number of lamb observations. Design effects and effective sample sizes were calculated separately for each pathogen and were used to quantify the potential reduction in independent information associated with within-herd clustering. These quantities were interpreted descriptively and were not used to replace the actual number of independent herd-level observations.

The ICC estimates were therefore considered in conjunction with the corresponding design effects and effective sample sizes when evaluating the hierarchical structure of the dataset. Because mortality outcomes were defined at the herd level, the potential consequences of clustering were further examined through a herd-level sensitivity analysis. The individual lamb was retained as the analytical unit for the primary RCCA, while the herd-level analysis was used to evaluate the robustness and directionality of the observed pathogen-mortality associations when the herd was treated as the independent observational unit.

ICCs were calculated using the ICC() function from the psych package in R version 4.4.3 in RStudio version 2026.05.0 (Build 218; “Golden Wattle” release) on Windows (Posit Software, PBC, Boston, MA, USA). For each pathogen, Cq values were arranged with herds as the observational units and the 20 lambs within each herd as repeated observations, and ICC(1) was used to quantify the proportion of variance attributable to between-herd differences. The complete analytical procedure and corresponding calculations are provided in Supplementary Materials S1.

#### 2.4.3. Herd-Level Sensitivity Analysis

To assess the robustness of the individual-level findings to the hierarchical structure of the data, a herd-level sensitivity analysis was performed using the 10 herds as the independent observational units. For each pathogen, individual-lamb Cq values were aggregated within herd by calculating the arithmetic mean across the 20 sampled lambs. The resulting herd-level mean Cq values were then related to the corresponding herd-level on-farm and feedlot mortality rates.

Pearson’s product–moment correlation coefficients were calculated between herd-level mean Cq values and each mortality outcome for each pathogen using the Correlation/Association test function available in the Correlation test menu of XLSTAT (Addinsoft Pearson Edition 2014, Paris, France). Because only 10 herds were available, this analysis was considered exploratory and was not intended to provide definitive inferential evidence. Instead, the magnitude and direction of the herd-level correlations were examined to determine whether the associations observed in the individual-level analysis were directionally consistent when the herd, rather than the individual lamb, was treated as the unit of analysis.

Because Cq values were entered directly into the analyses, with higher Cq values representing lower measured molecular signals, the direction of the herd-level correlations was interpreted accordingly. Thus, negative correlations between mortality and mean Cq values indicate that higher mortality was associated with lower mean Cq values, whereas positive correlations indicate that higher mortality was associated with higher mean Cq values. No transformation or inversion of Cq values was applied for this sensitivity analysis, consistent with the primary statistical analysis.

Given the limited number of independent herds (n=10), statistical significance was interpreted cautiously. The herd-level sensitivity analysis was considered complementary to the primary RCCA and was not intended to replace the individual-level analysis. Rather, it was performed to determine whether the direction and magnitude of the principal pathogen-mortality associations were maintained when the herd was treated as the independent observational unit.

#### 2.4.4. Regularized Generalized Canonical Correlation Analysis (RCCA)

RCCA was performed using the Canonical Correlation Analysis function available in the Multiblock Data Analysis menu of XLSTAT 2014 [34]. The multivariate relationship between the pathogen and mortality variable sets was additionally evaluated using the MANOVA framework implemented through the SPSS MANOVA syntax detailed in Supplementary Materials S2 and executed in SPSS version 25.0 [35]. The MANOVA procedure was used as a complementary multivariate inferential framework for evaluating the overall association between the pathogen and mortality variable sets, rather than as a conventional analysis of variance comparing predefined groups.

RCCA was used to assess the multivariate relationships between the pathogen and mortality variable sets while reducing the potential influence of multicollinearity and overfitting arising from correlated variables within each set. The individual lamb was retained as the analytical unit, consistent with the hierarchical assessment described above. This distinction was important because pathogen measurements were available at the individual-lamb level, whereas mortality outcomes were defined at the herd level.

Because the assumptions required for conventional parametric inference were not consistently satisfied, permutation-based inference was additionally used to evaluate the statistical significance of the multivariate associations. Conventional approximate F statistics and associated parametric probabilities obtained from the MANOVA procedure were retained for descriptive comparison, whereas permutation-derived empirical *p*-values were used as the primary basis for statistical inference.

For the lamb-level MANOVA, the 200 individual lambs were used as the analytical observations, and the multivariate statistics were calculated at the lamb level. The complete R code used for the MANOVA, including calculation of Wilks’ lambda, Pillai’s trace, the Lawley-Hotelling trace, Roy’s largest root, and the corresponding permutation distributions and empirical *p*-values, to be loaded in R version 4.4.3 in RStudio version 2026.05.0 (Build 218; “Golden Wattle” release) on Windows (Posit Software, PBC, Boston, MA, USA) is provided in Supplementary Materials S3.

For the RCCA and its permutation-based inference, the hierarchical structure of the data was explicitly taken into account. Because the mortality outcomes were defined at the herd level, herd was used as the exchangeable unit for the RCCA permutation procedure. The RCCA regularization parameters were first established through the RCCA reproduction procedure described in Supplementary Materials S4, in which the regularization parameter space was systematically searched to identify values that reproduced the canonical correlations of the original RCCA. The regularization parameters were subsequently evaluated a posteriori using 10-fold cross-validation, which identified λ_1_ = 0.9 and λ_2_ = 0.3; these values were then used as fixed inputs for the RCCA performed in XLSTAT 2014 and retained unchanged throughout the permutation procedure.

Specifically, 10,000 herd-block permutations were generated by jointly reassigning the paired on-farm and feedlot mortality outcomes among the 10 herds while maintaining all pathogen observations within their original herd. Thus, the 20 lamb-level pathogen observations belonging to each herd remained together throughout each permutation, whereas the paired on-farm and feedlot mortality outcomes were reassigned as a single herd-level block. This procedure preserved the within-herd dependence structure of the pathogen observations and the pairing between the two mortality outcomes while disrupting their association with the pathogen variables. The individual lamb therefore remained the analytical observation for the RCCA, whereas herd constituted the exchangeable unit for permutation-based inference.

For each permuted dataset, the RCCA was recalculated using the fixed regularization parameters. The resulting canonical correlations and associated global RCCA statistics, including Wilks’ lambda, Pillai’s trace, the Lawley-Hotelling trace, and Roy’s largest root, were used to generate the corresponding empirical null distributions. Empirical *p*-values were calculated by comparing the observed statistics with their respective herd-block permutation distributions. The complete R code used for the herd-block permutation-based RCCA, including calculation of the observed statistics, permutation distributions, 95% permutation intervals, and empirical *p*-values, is provided in Supplementary Materials S5.

Given that only 10 herds constituted the independent exchangeable units, the RCCA permutation-based inference was interpreted in the context of this limited number of independent clusters.

##### Pearson’s Product–Moment Correlations

Following the multicollinearity assessment, Pearson’s product–moment correlation coefficients were calculated to quantify linear relationships within and between the mortality parameters and the Cq variables corresponding to pathogens associated with the ovine respiratory complex. Correlations were generated using the Canonical Correlation Analysis function available in the Multiblock Data Analysis menu of XLSTAT 2014. Correlation coefficients were interpreted according to the criteria outlined by Gil Lebrero et al. [36], as cited by Olson [37].

Pairwise correlation analyses were performed primarily to characterize the correlation structure among variables and to inform subsequent multivariable analyses. As these analyses were exploratory and descriptive rather than a series of independent confirmatory hypothesis tests, adjustment for multiple comparisons was not considered necessary. Potential multicollinearity was assessed using variance inflation factors (VIF), and no problematic levels were detected.

##### Validity

Regularization of canonical correlation analysis is particularly useful in settings involving high dimensionality and limited sample sizes, as it reduces the risk of overfitting and unstable parameter estimates [38]. Previous studies have demonstrated that regularized canonical correlation methods can provide robust estimates of linear associations when their underlying assumptions are adequately addressed [39].

Following Olson’s guidance [37], Pillai’s trace was considered as a multivariate measure for evaluating the overall significance of the canonical relationships because of its relative robustness to departures from conventional multivariate assumptions, including non-normality and heterogeneity of variance–covariance matrices. Regularized canonical correlation analysis (RCCA) was used to characterize the multivariate relationships between the pathogen and mortality variable sets. Conventional multivariate statistics and their corresponding parametric significance tests were initially obtained using the Canonical Correlation function in the Multivariate Analysis menu and the MANOVA multivariate regression procedures implemented in Stata version 16 (StataCorp, College Station, TX, USA, 2017). These statistics were retained as descriptive measures and for comparison with the corresponding permutation distributions, whereas empirical *p*-values were derived from the herd-block permutation procedure described above.

Because the assumptions underlying conventional parametric inference were not consistently satisfied, permutation-based inference was used as the primary approach for statistical significance. The conventional multivariate statistics obtained from the RCCA were retained as descriptive measures and for comparison with the corresponding permutation distributions, whereas empirical *p*-values were derived from the herd-block permutation procedure described above. This permutation scheme preserved the herd-level clustering structure by treating herds, rather than individual animals, as the exchangeable units. Given that mortality outcomes were defined at the herd level and that only 10 herds were available, statistical inference was interpreted cautiously in the context of this limited number of independent clusters.

##### Variability Explanation

Subsequently, eigenvalues were calculated based on the product of the model matrix and the inverse of the error matrix. These were derived from the squared canonical correlations. The largest eigenvalue is calculated according to [33]. The relative sizes of eigenvalues indicate the proportion of variance in canonical variates explained by their respective canonical correlations, with the first eigenvalue being the largest and most informative.

##### Canonical Correlations

Canonical correlations, ranging from −1 to 1, were interpreted according to standard Pearson correlation criteria [29], following the guidelines of Olson [37], as cited by Gil-Lebrero et al. [36]. Values close to 0 indicate weak associations, whereas values approaching |1| indicate strong relationships. Canonical correlations of ≥0.30 were considered meaningful. The squared canonical correlation ($R_{c}^{2}$) was additionally calculated for each canonical function and interpreted as the proportion of variance shared between the corresponding canonical variates derived from the two variable sets. Thus, a canonical correlation of 0.30 corresponds to approximately 9% shared variance (approximately 10%), whereas larger canonical correlations indicate progressively greater shared variance between the pathogen and mortality canonical variates. Although it is common practice to report only the first canonical dimension, both canonical correlations were reported because they were interpretable components of the observed multivariate structure, following the recommendation of Marin Navas et al. [32].

Redundancy coefficients were also calculated to quantify the proportion of variance in one set of variables that could be explained by the canonical variates derived from the other set. Unlike squared canonical correlations, which quantify the variance shared between paired canonical variates, redundancy coefficients estimate the proportion of variance in the original variables of one set accounted for by the canonical variate derived from the opposite set.

For interpretation of the canonical functions, both standardized canonical coefficients (presented in equations, conforming the canonical structure of the model) and correlations between input variables and canonical variables (canonical loadings), represented as a biplot, were examined. The canonical structure and the corresponding standardized canonical functions are presented in Equations (1)–(4), which show the contribution of each input variable to the construction of the canonical variates. Standardized canonical coefficients represent the relative weights assigned to the original variables in constructing each canonical variate (Y1 and Y2), whereas canonical loadings (biplot) represent the correlations between each original variable and its corresponding canonical variate, indicating the strength and direction of its association with the underlying canonical dimension. Because canonical coefficients may be influenced by multicollinearity among variables, canonical loadings were given greater emphasis when interpreting the substantive meaning and relative importance of variables within each canonical function. Thus, canonical coefficients describe how each variate is constructed, whereas canonical loadings indicate which variables most strongly characterize the resulting canonical dimension.

##### Roots

Roots represent the ordered rank of each eigenvalue, from largest to smallest. The number of roots equals the number of variables in the smaller set (mortality parameters). Each root tests the null hypothesis that all associated canonical correlations are zero in the population. By examining each set of roots, the number of meaningful dimensions describing the relationships between variable sets can be determined. Typically, later roots correspond to smaller eigenvalues and contribute less explanatory power. Each Wilks’ lambda value is calculated as the product of (1 − canonical correlation) values for the tested set of canonical correlations. In this context, Wilks’ lambda acts as a multivariate analogue of R^2^. Its interpretation is inverse to R^2^. Smaller values reflect better performance of the discriminant function.

##### Canonical Correlation Analysis k-Fold Cross-Validation

To validate the RCCA results, 10-fold cross-validation was performed, minimizing the influence of sample-specific error [40,41]. The value of k = 10 was chosen based on its association with optimal performance and low bias in load estimation. This was implemented in R 4.1.1 for Windows, using RStudio Version 1.4.1717, through the CCA package by González et al. [42] and mixOmics 6.16.3 accessed via the R Project site (R: The R Project for Statistical Computing). Regularization parameters (λ_1_ and λ_2_) were estimated using the tune.rcc function [43], which iteratively selected positive values for λ_1_ and λ_2_ to identify those that produced the highest cross-validation (CV) score. Regularization parameters (λ_1_ and λ_2_) were evaluated using 10-fold cross-validation. The optimal values identified were λ_1_ = 0.9 and λ_2_ = 0.3. These values were subsequently used as fixed inputs for the RCCA performed in XLSTAT 2014 and were retained unchanged throughout the herd-block permutation procedure.

## 3. Results

### 3.1. Statistical Analysis

#### 3.1.1. A Priori Assumptions

The primary a priori assumptions underlying regularized canonical correlation analysis (RCCA) were carefully evaluated. Multivariate normality was assessed using both the Shapiro-Wilk and D’Agostino-Pearson tests. While the Shapiro-Wilk test suggested deviations from normality, the D’Agostino-Pearson test indicated no significant departure (*p* > 0.05), suggesting that normality could be reasonably assumed. Homoscedasticity was tested via Levene’s test, which indicated a statistically significant violation (*p* < 0.05); to address this, permutation tests—robust to heteroscedasticity—were implemented for inference, following best-practice recommendations. Outlier analysis using the ROUT method (Q = 1%) found no evidence of influential data points across the measured parameters, affirming the assumption of outlier absence. Additionally, sample size requirements were exceeded, and multicollinearity was ruled out through variance inflation factor (VIF) analysis, with all VIF values falling below the critical threshold of 5 (Supplementary Table S1, Figure 1). Collectively, these results support the validity of the RCCA despite minor deviations, thanks to the use of robust inferential methods.

#### 3.1.2. Assessment of the Within-Herd Hierarchical Structure of Pathogen Data

The assessment of the within-herd hierarchical structure showed ICC values ranging from 0.129 to 0.363 across the seven pathogens evaluated (Table S1). The lowest ICC was observed for *Mannheimia haemolytica* (ICC = 0.129), whereas the highest was observed for *Mesomycoplasma ovipneumoniae* (ICC = 0.363). Intermediate values were obtained for *Histophilus somni* (ICC = 0.143), *Bibersteinia trehalosi* (ICC = 0.149), PIV3 (ICC = 0.196), Orf virus (ICC = 0.261), and *Pasteurella multocida* (ICC = 0.350).

According to the commonly used descriptive classification proposed by Koo and Li [44], ICC values below 0.50 correspond to poor reliability. Under this classification, all seven ICC estimates fell below 0.50. Although this classification was originally developed for interpreting reliability rather than for determining whether a clustered study design requires statistical adjustment, it provides a descriptive indication that the observed between-herd component was not large relative to the total variance.

The potential impact of the observed clustering was further quantified using the design effect and effective sample size (Table S1). Design effects ranged from 3.442 for *M. haemolytica* to 7.899 for *M. ovipneumoniae*, corresponding to effective sample sizes ranging from 58.1 to 25.3 observations, respectively. *M. ovipneumoniae* and *P. multocida*, which were among the pathogens most closely associated with on-farm mortality, showed design effects of 7.899 and 7.649 and effective sample sizes of 25.3 and 26.1, respectively.

Thus, although the ICC values were below the 0.50 descriptive threshold, the corresponding design effects indicated that within-herd clustering could substantially reduce the amount of independent information relative to the nominal sample size of 200 lambs. Accordingly, the individual-lamb RCCA results were interpreted with consideration of the hierarchical structure, and a herd-level sensitivity analysis was performed using the 10 herds as the independent observational units.

#### 3.1.3. Herd-Level Sensitivity Analysis

The herd-level sensitivity analysis evaluated whether the principal pathogen-mortality associations were directionally maintained when the herd was considered the independent observational unit. Mean Cq values were calculated for each pathogen within each of the 10 herds and correlated with the corresponding herd-level mortality rates (Table 2).

For on-farm mortality, the correlation with *M. ovipneumoniae* mean Cq was negative (r = −0.466, *p* = 0.175), consistent in direction with the individual-level association (r = −0.2955). The association between *P. multocida* mean Cq and on-farm mortality was also negative (r = −0.048, *p* = 0.896), closely matching the individual-level estimate (r = −0.0491).

For feedlot mortality, positive correlations were observed with *M. haemolytica* (r = 0.839, *p* = 0.002), *P. multocida* (r = 0.472, *p* = 0.169), and PIV3 (r = 0.533, *p* = 0.113). These associations were directionally consistent with the corresponding individual-level correlations for *M. haemolytica* (r = 0.3226), *P. multocida* (r = 0.2667), and PIV3 (r = 0.2122).

Given the small number of independent herds (n = 10), the herd-level analysis was considered exploratory. Only one of the pathogen-mortality associations reached statistical significance at the herd level, namely the association between *M. haemolytica* and feedlot mortality (r = 0.839, *p* = 0.002), whereas the remaining associations were non-significant. Importantly, the herd-level analysis did not reveal a consistent pattern of associations indicating that accounting for herd as an additional analytical level would materially alter the principal individual-level findings. Therefore, the results of the sensitivity analysis supported retaining the individual lamb as the analytical unit for the primary RCCA, without incorporating herd as an additional level.

#### 3.1.4. Descriptive Analysis

At the individual level, high prevalences of *M. haemolytica* and *M. ovipneumoniae* (91.5% and 73.5%, respectively) were detected. In addition, *B. trehalosi* and *P. multocida* were detected in approximately 30.5% of the animals analysed. Other pathogens, such as *H. somni*, Orf virus and PIV3, were detected less frequently, with prevalence rates of 10.5%, 10.0% and 10.0%, respectively. When individual prevalences were compared according to the season of sampling (Table 1), a slight increase in virus prevalence was observed in winter, although this difference was not statistically significant (*p* > 0.05). At herd level, high prevalence rates of *P. multocida*, *M. haemolytica*, *B. trehalosi* and *M. ovipneumoniae* were detected in BAL samples (90–100%; Table 1). The prevalences of *H. somni*, Orf virus and PIV3 ranged between 50% and 60% at herd level (Table 1).

The distribution of pathogen combinations revealed a predominance of profiles involving *M. haemolytica* and *M. ovipneumoniae* (Table 3). The most frequent combination was *M. haemolytica* + *M. ovipneumoniae*, detected in 54 lambs (27.0%), followed by *M. haemolytica* alone (27, 13.5%) and *M. haemolytica* + *M. ovipneumoniae* + *P. multocida* (20, 10.0%). Other frequent profiles included *M. haemolytica* + *B. trehalosi* (14, 7.0%), *M. haemolytica* + *M. ovipneumoniae* + *B. trehalosi* (13, 6.5%), and *M. haemolytica* + *M. ovipneumoniae* + *P. multocida* + *B. trehalosi* (12, 6.0%). Less frequent combinations, involving PIV3, *H. somni*, and/or Orf virus, were detected in four or fewer lambs, each. Overall, the complete distribution comprised a broad range of pathogen profiles, including single infections and combinations of up to six pathogens, although most of the less common profiles occurred in ≤2% of the study population. Two lambs (1.0%) were negative for all pathogens analysed. The complete distribution of pathogen combinations is presented in Supplementary Table S2.

#### 3.1.5. Regularized Generalized Canonical Correlation Analysis (RCCA)

A detailed summary of the statistical measures used in the canonical correlation analysis, including their interpretation, relationship with the input variables, assessment of statistical and practical significance, and corresponding presentation within the manuscript, is provided in Supplementary Table S3.

##### Pearson’s Product–Moment Correlations

Significant correlations were observed among several microorganisms associated with the ovine respiratory complex (Figure 2). Because pathogen measurements were expressed as Cq values, these correlations describe the relationships between the corresponding Cq measurements. Notably, *M. ovipneumoniae* showed strong positive correlations with *M. haemolytica* (r = 0.5120) and *P. multocida* (r = 0.5272), indicating that their Cq values tended to vary in the same direction and suggesting a tendency for these pathogens to be detected concurrently within the sampled environments. *M. haemolytica* and *P. multocida* also displayed a moderate positive correlation (r = 0.4705), while PIV3 was significantly correlated with *P. multocida* (r = 0.4870), indicating a similar pattern of correlated detection. A weaker positive correlation was observed between *H. somni* and *M. haemolytica* (r = 0.2203), whereas *B. trehalosi* did not show significant associations with the other microorganisms, suggesting a comparatively more independent pattern of detection.

Regarding the relationship between pathogens and mortality rates (Figure 2), on-farm mortality showed a moderate negative correlation with the Cq values of *M. ovipneumoniae* (r = −0.2955). Because lower Cq values indicate higher pathogen loads, this negative correlation corresponds to an association between higher *M. ovipneumoniae* load and higher on-farm mortality. This relationship is consistent with the opposite positions of on-farm mortality and *M. ovipneumoniae* on the first canonical dimension. Specifically, *M. ovipneumoniae* showed a positive canonical loading on F1 (0.7754), whereas on-farm mortality showed a strong negative loading (−0.8841), indicating that these variables characterize opposite ends of the same canonical gradient. The positive standardized canonical coefficient for *M. ovipneumoniae* (0.8209) and the negative coefficient for on-farm mortality (−1.0235) should not be interpreted as pairwise correlations; rather, they represent the respective weights used to construct the canonical variates from their corresponding variable sets. Therefore, the apparent directional difference arises from comparing different statistical quantities rather than from a contradiction between the Pearson and RCCA results. The inverse association in Cq values may reflect the high prevalence of subclinical colonization in apparently healthy lambs, suggesting that pathogen detection does not necessarily correspond to active pathogenicity.

Regarding the relationship between pathogens and mortality rates (Figure 2), on-farm mortality showed a moderate negative correlation with the Cq values of *M. ovipneumoniae* (r = −0.2955). On-farm mortality showed a weak and non-significant correlation with the Cq values of *P. multocida* (r = −0.0491). In contrast, feedlot mortality was positively correlated with the Cq values of *M. haemolytica* (r = 0.3226), *P. multocida* (r = 0.2667), and parainfluenza virus type 3 (r = 0.2122). Mortality rates on-farm and in feedlots were also positively correlated (r = 0.2859). Because Cq values are inversely related to pathogen load, the positive correlations with feedlot mortality indicate that higher mortality rates tended to occur alongside higher Cq values, corresponding to lower measured loads of *M. haemolytica*, *P. multocida*, and PIV3.

##### Validity and Variability Explanation

Table 4 presents the multivariate test statistics obtained from the global MANOVA using the 200 individual lambs as the analytical observations. The four conventional multivariate criteria—Wilks’ lambda, Pillai’s trace, the Lawley-Hotelling trace, and Roy’s largest root—yielded statistically significant approximate F tests (all *p* < 0.0001), indicating a statistically detectable multivariate structure in the association between the set of pathogen detection variables and the two mortality outcomes (on-farm and feedlot mortality rates) at the lamb-observation level.

The permutation results presented in Table 4 correspond to the lamb-level MANOVA implemented in Supplementary Materials S3. The observed multivariate statistics were compared with their corresponding empirical null distributions generated from 10,000 permutations. The resulting empirical *p*-values were <0.0001 for Wilks’ lambda, Pillai’s trace, the Lawley-Hotelling trace, and Roy’s largest root.

Thus, both the conventional MANOVA and the corresponding lamb-level permutation analysis indicated a statistically detectable multivariate structure among the 200 lamb-level observations. These results should nevertheless be interpreted in light of the hierarchical organization of the dataset, because the mortality outcomes were defined at the herd level and the 200 lambs were distributed across only 10 herds. Accordingly, the MANOVA was retained as a lamb-level analysis describing the multivariate structure of the observed data, whereas the herd-level exchangeability structure was explicitly incorporated into the RCCA permutation analysis described below.

Importantly, the lamb remained the analytical observation for the MANOVA and RCCA, whereas herd was used as the exchangeable unit for the RCCA permutation procedure.

Regularized canonical correlation analysis (RCCA) was used to further characterize the multivariate relationship between the seven pathogen detection variables and the two mortality outcomes. The analysis was conducted using the 200 individual lambs as the analytical observations, while the herd structure was accounted for during permutation-based inference. The original RCCA identified two canonical dimensions, with canonical correlations of 0.4832 and 0.4192, respectively, corresponding to 23.35% and 17.57% shared variance between the two variable sets.

For inference, the RCCA was recalculated under a herd-block permutation scheme in which the 10 herds were treated as the exchangeable units. The paired on-farm and feedlot mortality outcomes were jointly reassigned among herds, while the pathogen observations within each herd remained together. A total of 10,000 permutations were performed, with the regularization parameters held fixed at the values used for the reproduced RCCA. The resulting empirical distributions for the global RCCA statistics are presented in Table 5.

The herd-block permutation analysis yielded a Wilks’ lambda of 0.63237, compared with a permutation null mean of 0.69982 (95% permutation interval: 0.53946–0.85793; empirical *p* = 0.21478). Pillai’s trace was 0.40905 (null mean = 0.32236; 95% permutation interval: 0.14651–0.51905; empirical *p* = 0.18598), while the Lawley-Hotelling trace was 0.51587 (null mean = 0.41503; 95% permutation interval: 0.16075–0.74963; empirical *p* = 0.24298). Roy’s largest root was 0.22492 (null mean = 0.23083; 95% permutation interval: 0.10337–0.36623; empirical *p* = 0.52675). Thus, none of the global RCCA test statistics showed evidence of a statistically significant multivariate association under the herd-block permutation procedure.

As with the MANOVA, these results indicate that the apparent multivariate structure identified at the lamb level should be interpreted in light of the limited number of independent herds. The herd-block permutation procedure preserves the lambs as the analytical observations while restricting inferential exchangeability to the 10 herd-level clusters.

##### Canonical Correlations, Structure, Standardized Coefficients and Loadings, Redundancy and Standard Roots

The canonical correlations were 0.4832 for the first canonical function (F1) and 0.4192 for the second canonical function (F2). Their corresponding squared canonical correlations were 0.2335 and 0.1757, indicating that 23.35% and 17.57% of the variance, respectively, was shared between the corresponding linear combinations of the pathogen and mortality variable sets. Thus, both canonical functions contributed to the overall multivariate relationship, with F1 showing a slightly stronger association between the two variable sets than F2. These values should be interpreted as shared variance between the canonical variates rather than as the proportion of variance in mortality directly explained by pathogen occurrence.

Interpretation of the canonical structure suggests that F1 represents a chronic colonization on-farm mortality axis, primarily characterized by positive associations with *M. ovipneumoniae* and *P. multocida* in opposition to on-farm mortality. In contrast, F2 represents an acute feedlot-associated mortality axis, characterized primarily by feedlot mortality and *M. haemolytica*, with additional positive associations with *P. multocida* and parainfluenza virus type 3. The canonical function F1 exhibited a canonical correlation of 0.4832, indicating a moderate association between the two sets of variables. However, this association was not statistically significant under the herd-block permutation procedure (empirical *p* > 0.05). F2 showed a slightly lower canonical correlation of 0.4192 and was likewise not statistically significant under the permutation-based inference.

Although neither canonical correlation was exceptionally high (i.e., >0.70), both canonical correlations exceeded the predefined descriptive threshold of 0.30 and were therefore retained for interpretation as components of the observed multivariate structure. They should not, however, be interpreted as statistically significant associations. F1 captured a stronger relationship between the variable sets than F2, consistent with its higher eigenvalue and its greater contribution to the total canonical eigenvalue (57.07% vs. 42.93%; Figure 3). Nevertheless, both canonical functions contributed substantially to the overall multivariate relationship and were therefore retained for interpretation.

Canonical correlations quantify the strength of the relationships between the paired canonical variates, whereas the corresponding canonical functions are defined by their standardized canonical coefficients, which indicate the relative weighting of each input variable in constructing F1 and F2. Accordingly, Equations (1)–(4) present the standardized canonical functions underlying these relationships: Equations (1) and (3) describe the pathogen-related variable set (Y1), whereas Equations (2) and (4) describe the mortality-related variable set (Y2). For F1, *M. ovipneumoniae* showed the strongest positive standardized coefficient in Y1 (0.8209), whereas on-farm mortality showed the strongest negative coefficient in Y2 (−1.0235). For F2, *M. haemolytica* showed the strongest positive coefficient in Y1 (0.8405), while feedlot mortality showed the strongest positive coefficient in Y2 (0.9226). These coefficients describe the mathematical construction of the canonical functions and should not be interpreted as independent causal effects.

F1Y1 = (−0.3629)(*H. somni*) + (−0.2647)(*B. trehalosi*) + (−0.1987)(*M. haemolytica*) + (0.8209)(*M. ovipneumoniae*) + (0.3418)(*P. multocida*) + (−0.0983)(Orfv) + (−0.4500)(PIV3). (1)

F1Y2 = (−1.0235)(On-farm Mortality) + (0.4877)(Feedlot Mortality).(2)

F2Y1 = (−0.1344)(*H. somni*) + (−0.4028)(*B. trehalosi*) + (0.8405)(*M. haemolytica*) + (−0.4805)(*M. ovipneumoniae*) + (0.2944)(*P. multocida*) + (0.0325)(Orfv) + (0.2939)(PIV3). (3)

F2Y2 = (0.2036)(On-farm Mortality) + (0.9226)(Feedlot Mortality).(4)

The standardized canonical coefficients describe how the canonical functions are mathematically constructed, whereas the canonical loadings, calculated as the correlations between the original input variables and their corresponding canonical variates, provide a more direct basis for interpreting the substantive meaning of each canonical dimension. For Y1, the F1 loadings were highest for *M. ovipneumoniae* (0.7754) and *P. multocida* (0.3732), whereas on-farm mortality showed a strong negative loading on F1 (−0.8841). Thus, F1 represents a multivariate gradient in which higher *M. ovipneumoniae* and *P. multocida* levels are associated with the positive end of the canonical dimension, while higher on-farm mortality is associated with its negative end. For F2, feedlot mortality showed the strongest positive loading (0.9808), followed by *M. haemolytica* (0.7422), *P. multocida* (0.5633), and parainfluenza virus type 3 (0.5582). These results indicate that F2 is primarily characterized by a feedlot mortality profile associated with these microorganisms. *M. ovipneumoniae* showed a weak positive loading on F2 (0.1823), despite having a negative standardized canonical coefficient (−0.4805) in Equation (3). This apparent discrepancy indicates that its negative coefficient reflects its conditional weighting within the multivariable canonical function, after accounting for the other microorganisms, rather than a strong negative association with F2. Therefore, when the sign of a standardized canonical coefficient differs from that of its corresponding loading, the loading is used primarily to determine the direction and substantive interpretation of the variable-function association.

Figure 4 displays a biplot of the canonical loadings, representing the correlations between the input variables and the canonical variables (Y1 and Y2), along the F1 and F2 axes. Together, these two dimensions account for 100% of the variance represented by the canonical solution. Two sets of variables are shown: the Y1 variables (red) represent the microbiological results from tracheobronchial washes, whereas the Y2 variables (green) represent on-farm and feedlot mortality rates.

On the F1 axis, which accounts for 57.07% of the variance, there is a clear separation between the microorganism and mortality variables. On-farm mortality shows a strong negative correlation with F1 (loading = −0.8841), whereas *M. ovipneumoniae* (0.7754) and *P. multocida* (0.3732) show positive loadings. This pattern indicates that the microorganism profile characterized by these positive-loading variables lies at the opposite end of the canonical gradient from on-farm mortality. The remaining Y1 variables show weaker negative or near-zero loadings, indicating more limited associations with F1.

The F2 axis, accounting for 42.93% of the variance, is strongly and positively correlated with feedlot mortality (loading = 0.9808) and *M. haemolytica* (0.7422). *P. multocida* (0.5633) and PIV3 (0.5582) also show moderate positive loadings, indicating their association with the same canonical dimension. In contrast, *B. trehalosi* shows a moderate negative loading (−0.3516), while *M. ovipneumoniae* has only a weak positive loading (0.1823). Consequently, F2 is best interpreted as an acute feedlot-associated mortality dimension, primarily characterized by feedlot mortality and *M. haemolytica*, with additional contributions from *P. multocida* and PIV3.

The clustering patterns observed in Figure 4 further support the distinction between the two canonical dimensions. The Y1 variables are relatively dispersed, with some positioned close to the origin and others showing stronger associations with the canonical axes, whereas the Y2 variables are more strongly aligned with specific directions. Variables positioned close to the origin have weaker correlations with the canonical dimensions and therefore contribute less to their interpretation. Conversely, variables located farther from the origin show stronger associations with the corresponding canonical axis. The relative proximity of variables to one another also provides an indication of similarity in their correlation patterns with the canonical dimensions. Furthermore, these spatial relationships should be interpreted as direct pairwise correlations given, they are supported by the corresponding Pearson correlation matrix (Figure 2).

For the dependent variable set Y1, the redundancy coefficients were 0.0332 for F1 and 0.0339 for F2, yielding a total redundancy of 0.0671. Thus, approximately 6.71% of the variance in Y1 is explained by the canonical variates derived from Y2, indicating a limited explanatory correspondence between the mortality variables and the microbiological variables. In contrast, the redundancy coefficients for Y2 were higher, at 0.0957 for F1 and 0.1037 for F2, yielding a total redundancy of 0.1994. Therefore, approximately 19.94% of the variance in Y2 is accounted for by the canonical variates derived from Y1. Although this represents a stronger explanatory correspondence than observed for Y1, the magnitude remains moderate, indicating that most of the variability in both variable sets is not captured by the canonical relationships.

The standardized canonical coefficients and canonical loadings therefore provide complementary information. The coefficients describe the relative weighting of the original variables in constructing each canonical function, whereas the loadings describe the strength and direction of their overall association with the resulting canonical dimension. In the present analysis, the agreement between the signs and relative magnitudes of the coefficients and loadings for the principal variables supports the interpretation of F1 as a *M. ovipneumoniae*–*P. multocida* versus on-farm mortality gradient and F2 as a *M. haemolytica*–feedlot mortality gradient. Importantly, these patterns represent multivariate associations between microorganism and mortality profiles and should not be interpreted as evidence of direct causal effects. The relatively low redundancy coefficients further indicate that, although the canonical solution identified structured multivariate relationships between the two variable sets, the canonical functions explained only a limited proportion of the total variability in the original variable sets.

##### Wilks’ Lambda and R2

The conventional Wilks’ lambda statistics were retained as descriptive measures of the canonical structure. For the first and second canonical functions, Wilks’ lambda values were 0.6318 and 0.8243, respectively, with the lower value for F1 indicating a stronger canonical relationship in the conventional formulation. The corresponding conventional F statistics were 7.0408 for F1 and 6.8191 for F2. Because the assumptions underlying parametric inference were not consistently satisfied and the herd-block permutation procedure provided the primary inferential framework, these conventional statistics and associated parametric *p*-values were not used as the basis for statistical significance. Under the herd-block permutation procedure, none of the global RCCA statistics reached an empirical *p*-value below 0.05 (Table 5). Accordingly, the canonical dimensions and their associated loadings are interpreted as descriptive characteristics of the multivariate structure rather than as independently significant effects.

##### Regularized Canonical Correlation Analysis k-Fold Cross-Validation

The results of the 10-fold RCCA cross-validation are presented in Figure 5. The cross-validation results were consistent with the canonical structure obtained from the full dataset, indicating good agreement between the cross-validated and full-sample results and suggesting that the observed canonical relationships were not substantially driven by sample-specific error. The regularization procedure was used to identify the optimal values of the regularization parameters λ_1_ and λ_2_ through 10-fold cross-validation. A regular grid of 100 × 100 candidate parameter combinations was evaluated within the ranges 0.001 < λ_1_ < 1 and 0.001 < λ_2_ < 1. The optimal combination was λ_1_ = 0.9 and λ_2_ = 0.3, corresponding to the white region in Figure 5. These values were subsequently fixed for the RCCA and retained unchanged throughout the permutation procedure used to obtain the empirical *p*-values reported in Table 5.

In practical terms, regularization limits the influence of random or sample-specific variation, helping the model identify more stable relationships between the two sets of variables. The selected values indicate stronger regularization of the pathogen variable set relative to the mortality variable set within the parameterization used for the RCCA. Because the interpretation of the regularization parameters depends on the specific implementation, their values were interpreted primarily in relation to the cross-validation criterion rather than according to an arbitrary threshold. The selected combination was therefore retained for the final RCCA and permutation analyses.

The optimal cross-validation score was approximately 0.404 (the lightest coloured level in the scale). The choice of dimensions d (1 ≤ d ≤ *p*) to include in the further analysis was performed according to González et al. [42], who suggests an empirical approach based on the inspection of the plot of canonical correlations versus dimensions and on the selection of the appropriate number of dimensions before a clear gap among canonical correlations. The selected regularization parameters were retained for the final RCCA analysis. The resulting canonical solution comprised two canonical dimensions, with correlations of 0.4832 and 0.4192, respectively (Table 4).

This means that the best performing model (based on cross-validation) uses a relatively strong penalization for X (Cq) and a moderate penalization for Y (Mortality). Practically, this means the model performs best when it selects fewer variables in X (via a stronger λ_1_). On the other hand, it does not need to be as aggressive in shrinking Y (because Mortality is likely a univariate response). This implies there is a good combination of parameters to use when fitting the final model.

## 4. Discussion

The control of the ORC is a major challenge for the veterinarian technicians of lamb production. The plurietiological nature, with the participation of different opportunistic pathogens, commonly found in the upper respiratory tract of healthy animals, hinders the implementation of specific sanitary measures [21]. In this work, we analysed the possible association between the presence of the main respiratory pathogens in BAL and the mortality rates recorded in sheep meat herds and feedlots.

Among the 200 BAL samples analysed by real-time PCR, only 2 (1%) were negative for all the respiratory pathogens investigated. A high prevalence of *M. haemolytica* (91.5%) and *M. ovipneumoniae* (73.5%) was detected, followed by *P. multocida* and *B. trehalosi* (30.5% respectively). High detection rates were expected given the opportunistic nature and frequent upper respiratory tract carriage of the Pasteurellaceae species, together with the widespread distribution of *M. ovipneumoniae* as an important predisposing agent in ORC [3,7,45]. The presence of other agents associated with respiratory disorders, including *H. somni* (10%), PIV3 (10.5%), and Orf virus (10%), has also been investigated; however, their prevalence was lower, and these agents were generally detected as part of co-infections.

Seasonal variation in pathogen prevalence has previously been suggested [24]. Although a higher prevalence was detected for PIV3 and Orf virus in winter compared to summer, no statistically significant seasonal differences were observed in our study (*p* > 0.05). Animals can be asymptomatic carriers of these pathogens, when exposed to appropriate temperature and humidity conditions, the transmission and multiplication of these pathogens in susceptible animals could allow the development of clinical disease and could explain the increased tissues concentration detected in other studies [46].

In our study, respiratory pathogens were frequently detected either individually or in combination. *M. haemolytica* was the most frequently detected pathogen alone (14%), while the combinations *M. haemolytica-M. ovipneumoniae* (25.50%) and *M. haemolytica-P. multocida-M. ovipneumoniae* (10%) were the most frequently detected. These data are interesting because, although molecular co-detection in asymptomatic animals does not imply active disease, the literature indicates that the most severe clinical presentations and lesions are often associated with mixed infections. [3,19,47,48].

The high prevalence of opportunistic pathogens of the family Pasteurellaceae and *M. ovipneumoniae* in the nasopharynx of apparently healthy sheep has been shown and, according to other studies, the severity of the respiratory disorders related to the ORC is significantly higher in lambs than in adults, which could be explained by the exposure to predisposing factors [24]. However, to date, it has not been possible to associate mortality rates during the stay of animals in livestock or feedlots with the presence of these pathogens.

The RCCA has proved to be a useful tool to assess possible associations and to determine the strength of these associations [22]. In the present study *M. ovipneumoniae* and *P. multocida* showed the closest associations with on-farm mortality. Comparable evidence from respiratory disease studies supports the clinical relevance of these pathogens, although direct comparisons across host species and production systems should be made cautiously. *P. multocida* or *M. ovipneumoniae* could act as a primary agent [14,49,50], producing the damage of the upper respiratory tract and predispose to the development of the pathology at early ages. *M. ovipneumoniae* is known to impair mucociliary clearance and induce chronic epithelial inflammation, facilitating secondary bacterial colonization. Similarly, *P. multocida* possesses capsular and adhesin-mediated mechanisms that enhance immune evasion and tissue persistence. The combined presence of these agents may therefore establish a subclinical inflammatory state that predisposes lambs to increased mortality under additional stressors.

The inverse association between *M. ovipneumoniae* Cq values and on-farm mortality may reflect the high prevalence of subclinical colonization in apparently healthy lambs, indicating that pathogen detection does not necessarily correspond directly to active pathogenicity. The observed association may also reflect differences in host susceptibility, pathogen–pathogen interactions, or the timing of sampling relative to disease development. Although the present analysis cannot establish causality or distinguish among these mechanisms, it suggests that pathogen-mortality relationships may occur within a complex respiratory environment in which molecular detection does not necessarily indicate active disease.

Importantly, the RCCA loadings describe the contribution of each variable to the corresponding canonical dimensions and should not be interpreted as independent effects or causal predictors of mortality. In particular, the herd-block permutation analysis did not provide significant global empirical *p*-values; therefore, individual pathogen loadings are interpreted as descriptive components of the multivariate canonical structure rather than as independently significant mortality effects.

The first canonical dimension was characterized by strong positive loadings for *M. ovipneumoniae* and *P. multocida* on the pathogen side and an opposing loading for on-farm mortality. Thus, these pathogens contributed most strongly to the multivariate structure represented by the first canonical dimension.

On the other hand, at the feedlot stage, *M. haemolytica* showed the strongest pathogen-side loading within the second canonical dimension, which was characterized primarily by feedlot mortality. This multivariate pattern is consistent with previous reports describing *M. haemolytica* as an important causative agent of acute respiratory disease and mortality in feedlot lambs, particularly under stress-associated conditions. However, given the non-significant herd-block permutation results, this finding should be interpreted as part of the canonical multivariate structure rather than as evidence of an independent effect of *M. haemolytica* on mortality [3,7,15,47]. The association observed in the present study is therefore biologically plausible, particularly considering the potential effects of transportation, regrouping, and other feedlot-related stressors on host susceptibility and respiratory disease. However, the interpretation of the Cq values requires particular caution. Because Cq values are inversely related to the amount of target nucleic acid detected, a positive association between mortality and Cq values does not indicate that higher mortality was associated with greater *M. haemolytica* molecular load. Rather, it indicates an association with higher Cq values, corresponding to lower measured molecular signals. This apparently counterintuitive pattern may reflect the fact that BAL samples were obtained from apparently healthy lambs and that molecular detection does not necessarily indicate active disease or pathogenic involvement.

More broadly, the positive associations between the Cq values of *M. haemolytica* and the feedlot mortality rate, together with the associations observed for *P. multocida* and PIV3, indicate that higher mortality rates were associated with higher Cq values for these microorganisms, corresponding to lower measured molecular signals. This pattern may have several non-mutually exclusive biological explanations. Relatively low pathogen loads may still be associated with disease in susceptible animals, particularly for microorganisms with substantial pathogenic potential. Alternatively, these microorganisms may occur as secondary or subclinical infections in disease processes involving other agents. Differences in pathogenicity, host susceptibility, pathogen–pathogen interactions, microbial competition, host adaptation, and the timing of sampling relative to disease development may all contribute to the observed associations. Although the present correlations cannot establish causality or distinguish among these mechanisms, they emphasize that pathogen-mortality relationships cannot be interpreted solely in terms of measured pathogen burden.

Similarly, *P. multocida* and PIV3 were associated with feedlot mortality, while *M. ovipneumoniae* also contributed to the feedlot canonical structure. These associations should likewise not be interpreted solely in terms of pathogen burden, given that the molecular measurements represent semi-quantitative Cq signals rather than absolute pathogen concentrations. The observed associations may instead reflect differences in pathogen occurrence, interactions among respiratory microorganisms, host susceptibility, or environmental and management conditions characteristic of the feedlot stage. The involvement of PIV3 in ovine respiratory disease has been reported previously, including evidence of viral detection and associated respiratory lesions; however, its contribution should be interpreted within the multifactorial nature of ORC rather than as an isolated cause of mortality [15,51,52,53].

Finally, the positive correlation between on-farm and feedlot mortality may indicate the influence of shared environmental or management factors across production stages. Thus, the overall pattern suggests that the relationship between respiratory pathogen detection and mortality is complex and cannot be inferred from pathogen load alone.

Overall, these findings should be interpreted within the epidemiological complexity of ORC, the expression of which varies among farms and feedlots and is strongly influenced by host, environmental, and production-system factors [53]. Consequently, respiratory health management strategies should consider not only the pathogens detected, including *M. haemolytica*, *P. multocida*, PIV3, and *M. ovipneumoniae*, but also predisposing management and environmental factors. Transportation and other stress-related management practices have been recognized as important contributors to respiratory disease [11,54,55]. In addition, alterations in the respiratory microbiota have been associated with respiratory disease, although evidence supporting this relationship has been more extensively documented in cattle [56].

This study has several limitations. First, pathogen detection was based on BAL samples obtained from apparently healthy lambs, which may not fully represent pathogen load during clinical disease. Second, Cq values reflect the molecular signal of the target nucleic acid rather than absolute pathogen concentration, viability, or virulence. Because BAL recovery volumes may vary among animals and Cq values were not normalized to a host reference gene or to the recovered BAL volume, differences in sample dilution may have influenced the Cq values. Therefore, these values should be interpreted as semi-quantitative molecular signals rather than absolute measures of pathogen load. Third, mortality data were aggregated at herd level, limiting individual-level inference. In addition, BAL samples were collected during 2023, whereas mortality rates were calculated from records recovering 2022–2023; therefore, the pathogen profiles detected in 2023 may not fully represent those present throughout the entire mortality-recording period. Finally, the observational design prevents causal conclusions, and associations should be interpreted within an epidemiological framework. Overall, the relationship between respiratory pathogens and mortality differed across production stages. *M. ovipneumoniae* and *P. multocida* were more closely associated with on-farm mortality, whereas *M. haemolytica* showed a stronger association during fattening. These stage-specific patterns may help guide targeted management and biosecurity measures at each production phase, improving ORC control, reducing losses, and supporting more rational antimicrobial use.

## 5. Conclusions

This study demonstrates that pathogen-mortality relationships within the ovine respiratory complex differed according to the production stage. *M. ovipneumoniae* and *P. multocida* showed the strongest positive loadings on the pathogen side of the first canonical dimension, which was characterized by an opposing loading for on-farm mortality, whereas *M. haemolytica*, together with *P. multocida* and PIV3, contributed most strongly to the second canonical dimension, which was characterized primarily by feedlot mortality. However, the herd-block permutation analysis did not provide significant global empirical *p*-values, and these canonical patterns should therefore be interpreted as descriptive components of the multivariate structure rather than as evidence of independent pathogen effects on mortality. The canonical structure showed limited redundancy, with approximately 19.94% total redundancy for the mortality variable set, indicating that most of the variability in the original mortality variables was not captured by the canonical relationships. Future studies integrating molecular pathogen surveillance with clinical, pathological, environmental, and management data, together with evaluations of preventive interventions, are needed to clarify these multifactorial relationships and their relevance to mortality. Such an integrated approach may contribute to improved animal welfare and reduced mortality losses in sheep production.

## Figures and Tables

**Figure 1:**
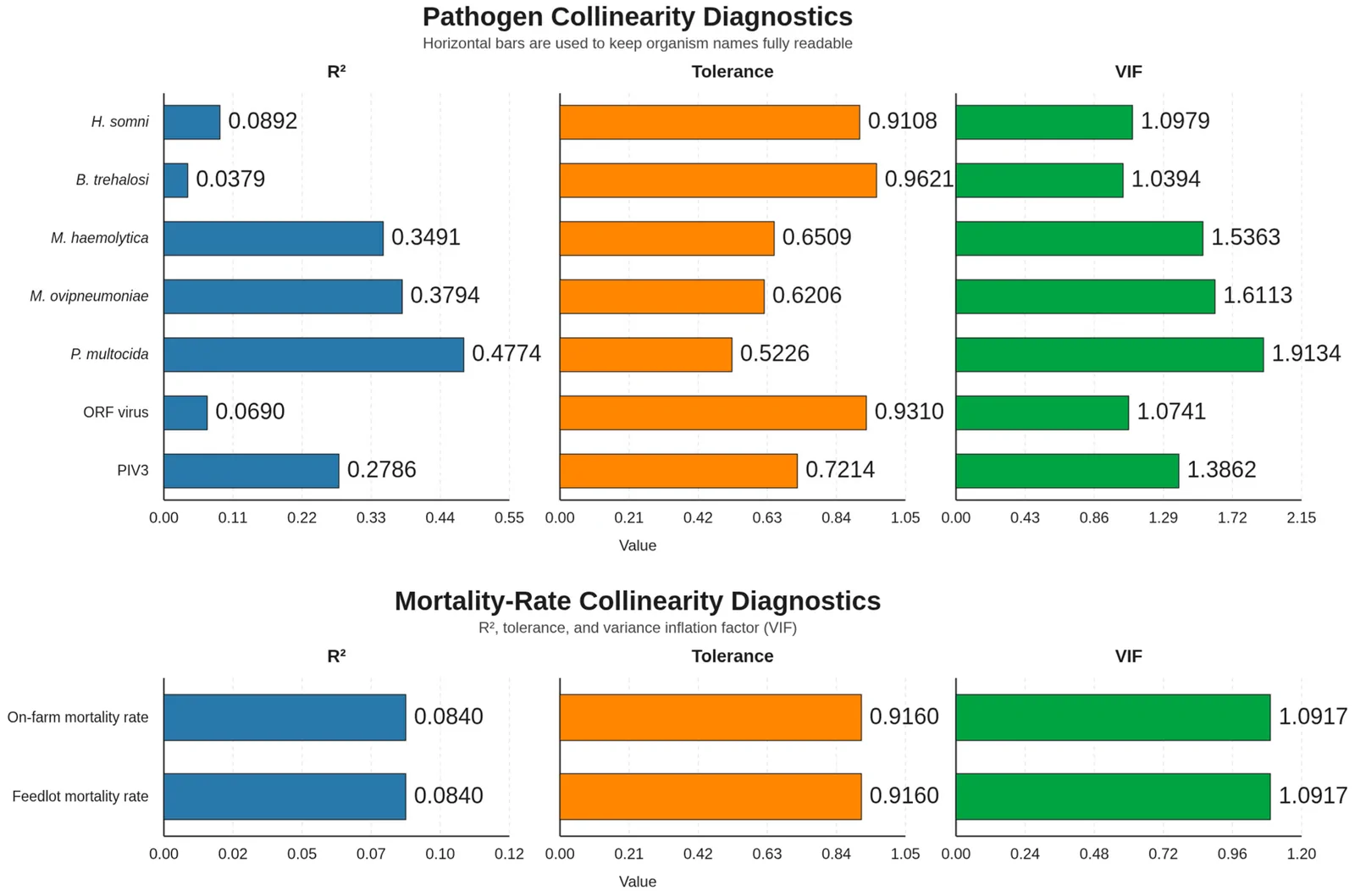
Collinearity diagnostics for the pathogen and mortality-rate variable sets included in the regularized canonical correlation analysis (RCCA).

**Figure 2:**
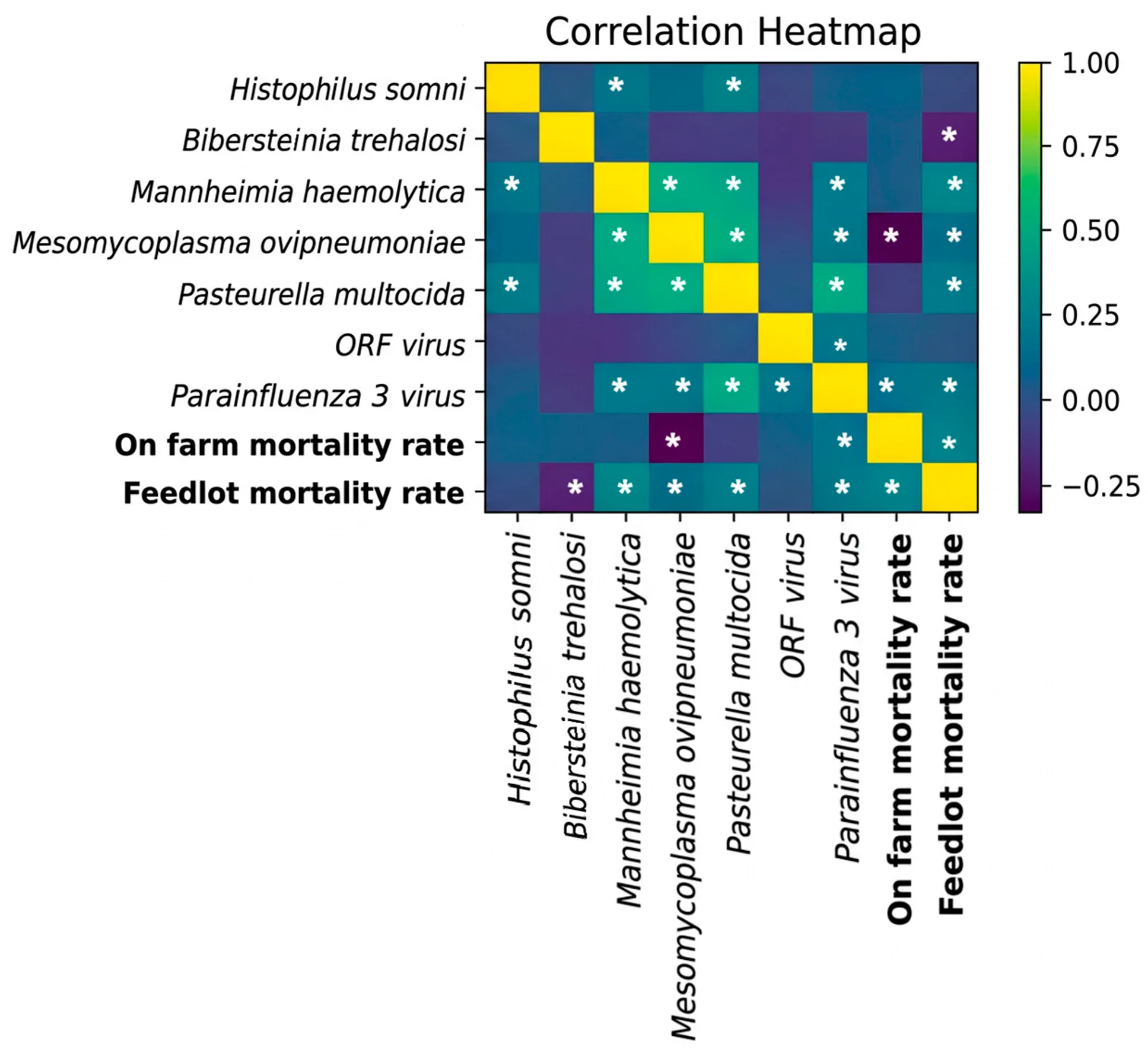
Pearson’s correlations between mortality related variable pairs and ovine respiratory complex related pathogens Cq variables. Color scale ranges from yellow (maximum positive value) to blue (maximum negative value). * *p* < 0.01.

**Figure 3:**
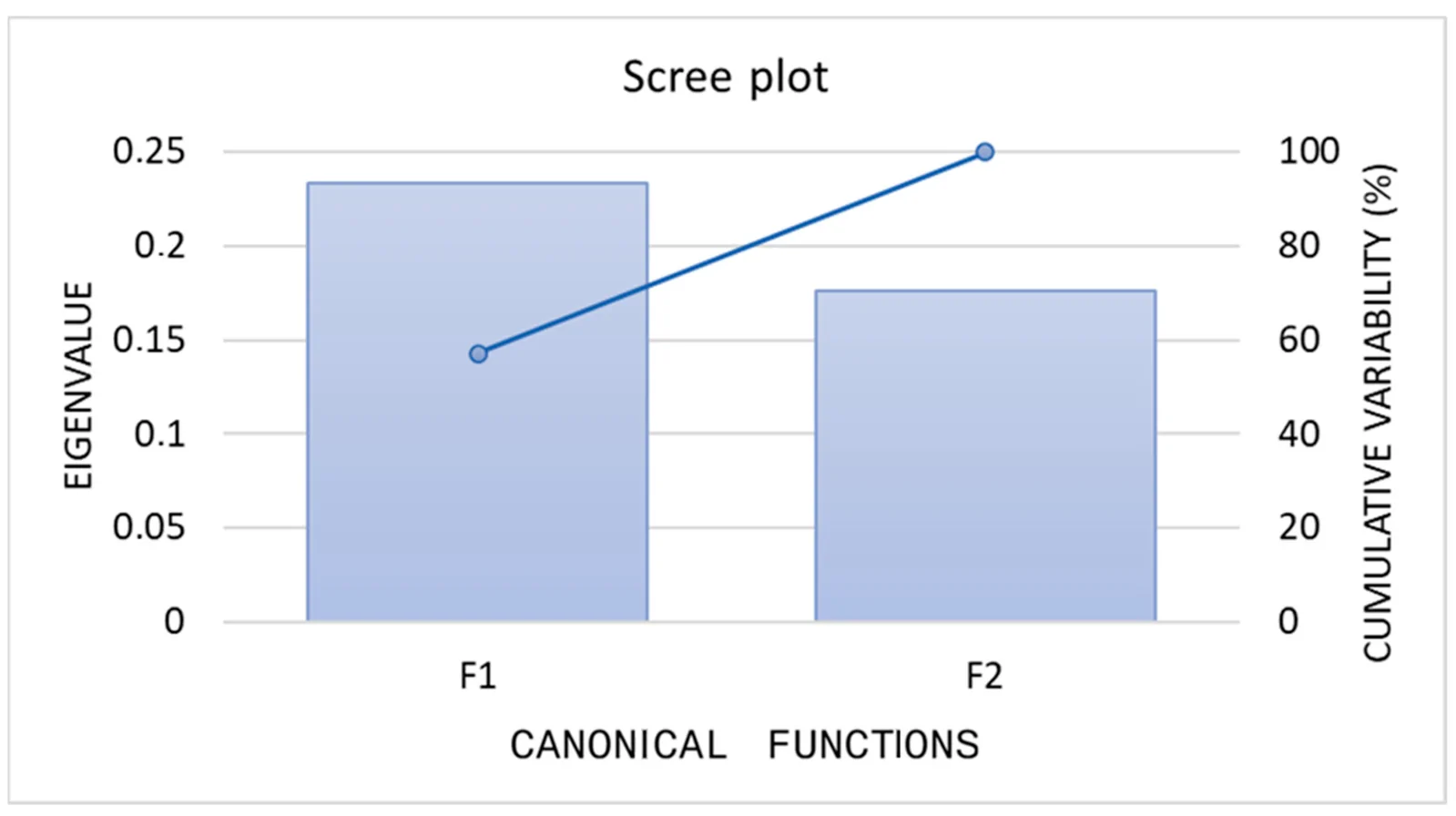
Eigenvalues and explained variability across dimensions identified by regularized canonical correlation analysis.

**Figure 4:**
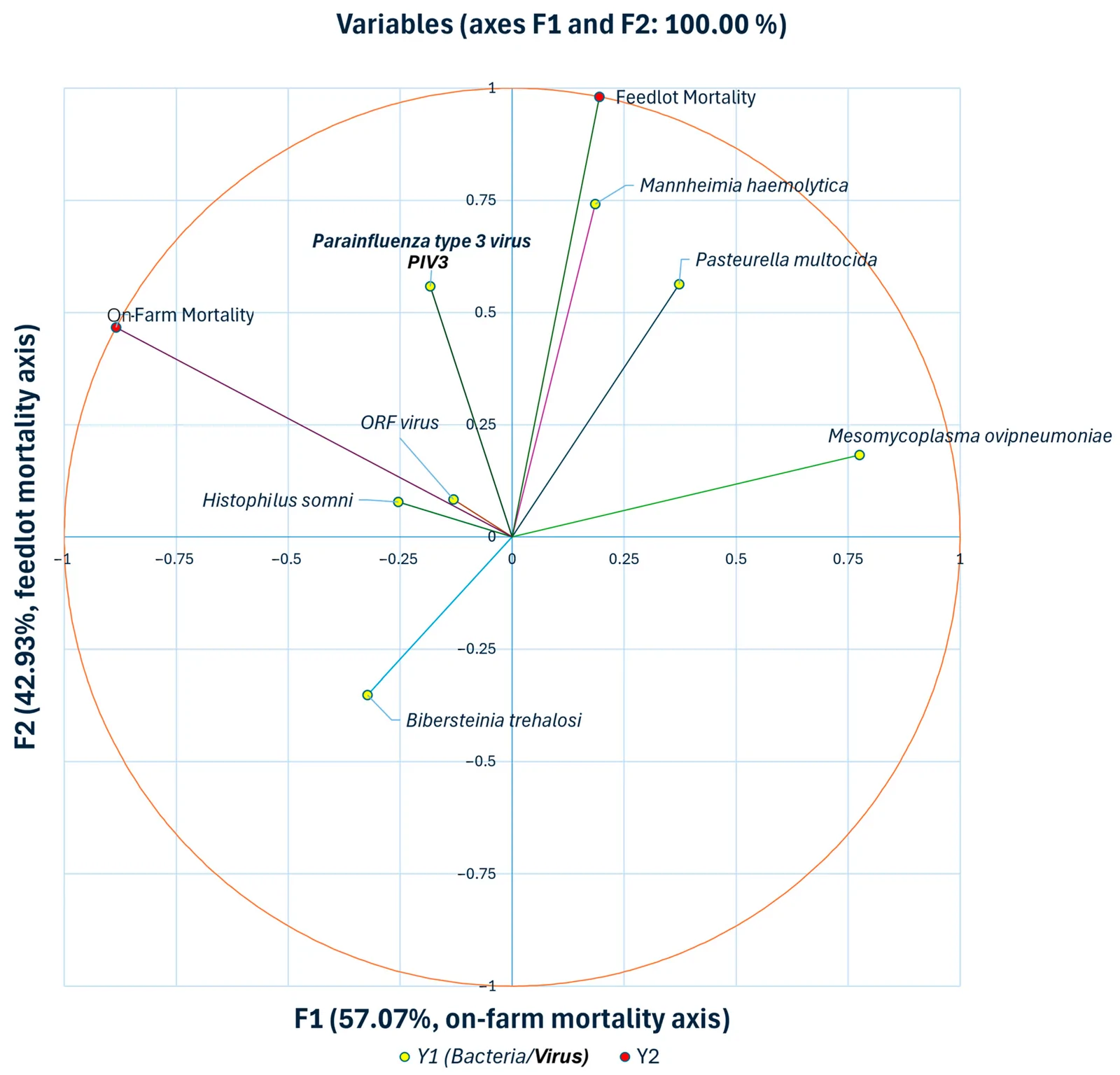
Biplot representing canonical loadings, or correlations between input variables and canonical variables (Y1 and Y2), along the F1 and F2 axes.

**Figure 5:**
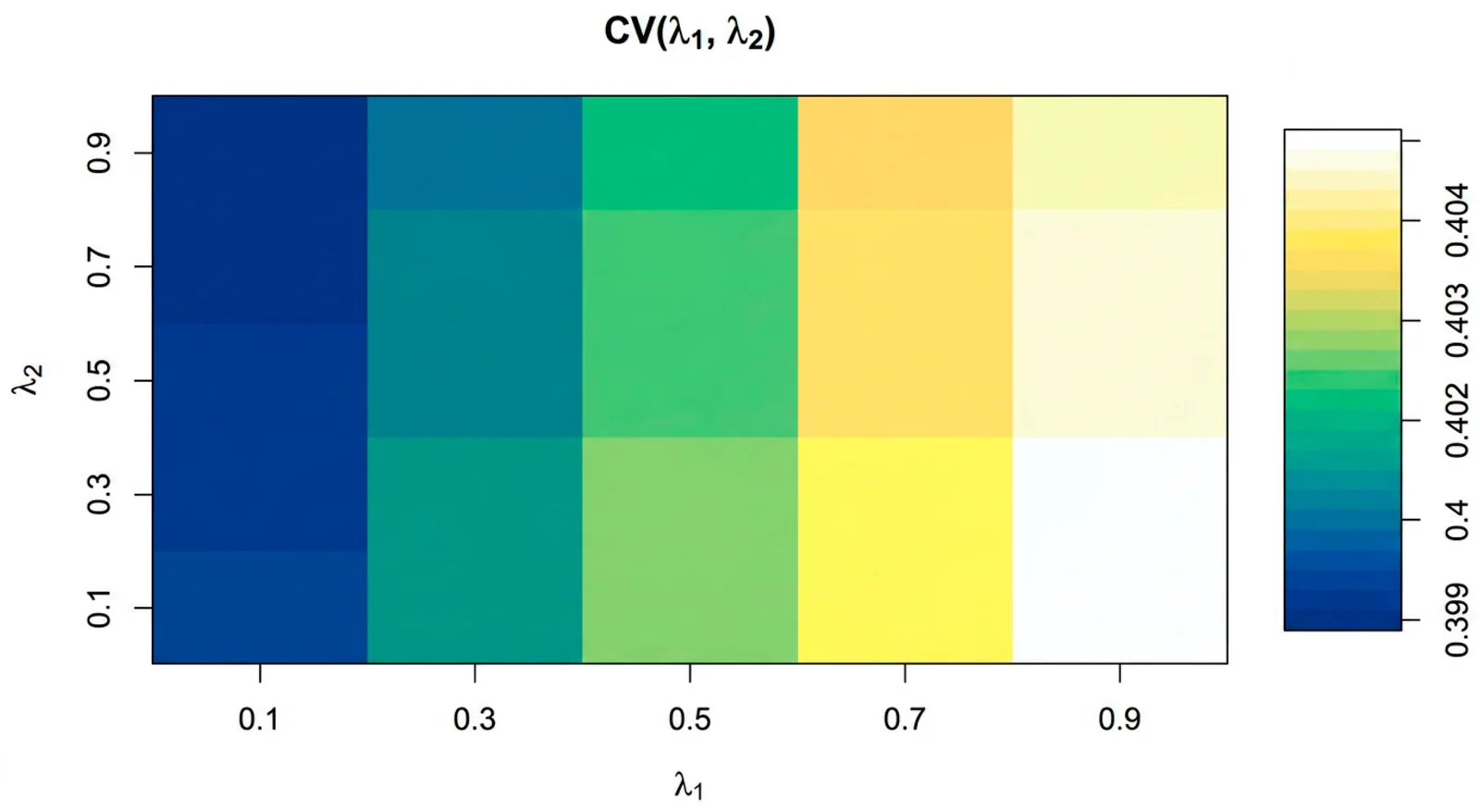
Optimal cross-validation scores for the values of the parameter of regularization (λ_1_ and λ_2_).

**Table 1:** Prevalence of pathogens detected in bronchoalveolar lavages (BAL) from 200 lambs belonged to 10 sheep meat farms expressed as % (95% CI).

Pathogen	Test	Herd Prevalence * (n = 10)	Individual Prevalence (n = 200)	Winter Prevalence ** (n = 80)	Summer Prevalence ** (n = 120)
*Mannheimia haemolytica*	ManHae-dtec-qPCR-F100 + IC	100%	91.5% (86.8–94.6%)	88.8% (80.0–94.0%)	93.3% (87.4–96.6%)
*Mesomycoplasma ovipneumoniae*	MycOvi-dtec-qPCR-F100 + IC	100%	73.5% (67.0–79.1%)	76.3% (65.9–84.2%)	71.7% (63.0–79.0%)
*Pasteurella multocida*	PasMul-dtec-qPCR-F100 + IC	90%	30.5% (24.5–37.2%)	33.8% (24.3–44.6%)	28.3% (21.0–37.0%)
*Bibersteinia trehalosi*	BibTre-dtec-qPCR-F100 + IC	100%	30.5% (24.5–37.2%)	28.8% (20.0–39.5%)	31.7% (24.0–40.4%)
*Histophilus somni*	HisSom-dtec-qPCR-F100 + IC	50%	10.0% (6.6–14.9%)	11.3% (6.0–20.0%)	9.2% (5.2–15.7%)
Orf virus	Orf-dtec-qPCR-F100 + IC	50%	10.0% (6.6–14.9%)	15.0% (8.8–24.4%)	6.7% (3.4–12.6%)
Parainfluenza 3 virus (PIV3)	EXOone Small ruminant Parainfluenza 3	60%	10.5% (7.0–15.5%)	15.0% (8.8–24.4%)	7.5% (4.0–13.6%)

* A farm is considered positive when at least one animal was positive. ** No significant seasonal differences (*p* > 0.05).

**Table 2:** Herd-level sensitivity analysis of the associations between mean pathogen Cq values and mortality rates across the 10 herds.

Pathogen	On-Farm Mortality r	*p* Value	Feedlot Mortality r	*p* Value
*H. somni*	0.294	0.410	0.016	0.966
*B. trehalosi*	0.154	0.671	−0.424	0.222
*M. haemolytica*	0.200	0.579	0.839	0.002
*M. ovipneumoniae*	−0.466	0.175	0.256	0.475
*P. multocida*	−0.048	0.896	0.472	0.169
Orf virus	0.132	0.715	0.038	0.916
PIV3	0.501	0.140	0.533	0.113

PIV3: Parainfluenza virus type 3.

**Table 3:** Most frequent pathogen combinations detected in bronchoalveolar lavage (BAL) samples from lambs. Pathogens within each combination are ordered according to their overall individual prevalence.

Species Identified in BAL *	Nº Samples (%)
*M. haemolytica*	27 (13.5%)
*M. haemolytica* + *M. ovipneumoniae*	54 (27.0%)
*M. haemolytica* + *B. trehalosi*	14 (7.0%)
*M. haemolytica* + *M. ovipneumoniae* + *B. trehalosi*	13 (6.5%)
*M. haemolytica* + *M. ovipneumoniae* + *P. multocida*	20 (10.0%)
*M. haemolytica* + *M. ovipneumoniae* + *P. multocida* + *B. trehalosi*	12 (6.0%)
*M. haemolytica* + *M. ovipneumoniae* + *P. multocida* + PIV3 **	5 (2.5%)
*M. haemolytica* + *M. ovipneumoniae* + *P. multocida* + *B. trehalosi* + *H. somni*	5 (2.5%)

Two BAL samples (1%) resulted negative to all pathogens analysed by qPCR. Only pathogen combinations detected in ≥5 samples are presented. Values are expressed as the number of samples and percentage of the total study population (n = 200). The complete list of pathogen combinations, including those detected in fewer than five samples, is presented in Supplementary Table S2. * Bronchoalveolar lavage. ** Parainfluenza virus type 3.

**Table 4:** Global permutation-based multivariate analysis of variance (MANOVA) examining the joint association of pathogen detection variables (*M. haemolytica*, *P. multocida*, *B. trehalosi*, *M. ovipneumoniae*, *H. somni*, Orf virus, and PIV3) with on-farm and feedlot mortality rates using 200 lambs as the analytical observations.

Statistic	Observed Value	df1 *	df2 **	F	Parametric *p*	Permutation Null Mean	95% Permutation Interval	Empirical *p*-Value
Wilks’ lambda	0.61117	14	382	7.61667	<0.0001	0.93044	0.87365–0.97103	0.0001
Pillai’s trace	0.43583	14	384	7.64261	<0.0001	0.07063	0.02917–0.12974	0.0001
Lawley-Hotelling trace	0.55931	14	196	7.83030	<0.0001	0.07437	0.02965–0.14139	0.0001
Roy’s largest root	0.31572	7	186	8.38923	<0.0001	0.05439	0.02057–0.10834	0.0001

* df1: numerator degrees of freedom; ** df2: denominator degrees of freedom.

**Table 5:** Herd-block permutation-based inference for the regularized canonical correlation analysis (RCCA) examining the multivariate association between pathogen detection variables and on-farm and feedlot mortality rates.

Statistic	Observed Value	Permutation Null Mean	95% Permutation Interval	Empirical *p*-Value
Wilks’ lambda	0.63237	0.69982	0.53946–0.85793	0.21478
Pillai’s trace	0.40905	0.32236	0.14651–0.51905	0.18598
Lawley-Hotelling trace	0.51587	0.41503	0.16075–0.74963	0.24298
Roy’s largest root	0.22492	0.23083	0.10337–0.36623	0.52675

## Data Availability

The datasets generated during and/or analyzed during the current study are available from the corresponding author on reasonable request.

## Supplementary Materials

The following supporting information can be downloaded at https://www.mdpi.com/article/10.3390/ani16193028/s1, Table S1: Assessment of the within-herd hierarchical structure of pathogen data; Table S2: Distribution of pathogen combinations detected in bronchoalveolar lavage (BAL) samples from 200 lambs; Table S3: Summary of the statistical measures used to evaluate the canonical structure, including their relationship with the original input variables, analytical meaning, interpretation, practical significance, and location within the manuscript; Material S1: R code used to calculate intraclass correlation coefficients (ICCs) for the assessment of the within-herd hierarchical structure of pathogen data; Material S2: SPSS MANOVA syntax; Material S3: R code and complete computational output for the global lamb-level permutation-based MANOVA; Material S4: R code for reproduction of the original RCCA and systematic search of the regularization parameters; Material S5: R code and complete computational output for the herd-block permutation-based RCCA.

## Abbreviations

The following abbreviations are used in this manuscript:

BAL	Bronchoalveolar lavage
BRD	Bovine respiratory disease
CCA	Canonical correlation analysis
Cq	Quantification cycle
CV	Cross-validation
df	Degrees of freedom
DNA	Deoxyribonucleic acid
FDR	False discovery rate
F1, F2	First and second canonical functions
95% CI	95% confidence interval
MANOVA	Multivariate analysis of variance
ORC	Ovine respiratory complex
PCR	Polymerase chain reaction
PIV3	Parainfluenza virus type 3
qPCR	Quantitative real-time polymerase chain reaction
RCCA	Regularized canonical correlation analysis
VIF	Variance inflation factor
