# Supplementary material for: Stage-Specific Associations Between Respiratory Pathogens and Mortality in Sheep: A Multivariate Analysis
Source: Animals (Basel). 2026 Sep 26;16(19):3028. doi: 10.3390/ani16193028 (PMC13663387; doi:10.3390/ani16193028)
Supplement: Supplementary file 1 [file animals-16-03028-s001.zip › Table S1.pdf]

Table S1. Assessment of the within-herd hierarchical structure of pathogen data

Pathogen	Between-herd variance	Within-herd variance	ICC(1)	Design effect	Effective sample size
Histophilus somni	0.3736	2.2387	0.143	3.717	53.8
Bibersteinia trehalosi	2.7224	15.4934	0.149	3.840	52.1
Mannheimia haemolytica	4.4402	30.1127	0.129	3.442	58.1
Mesomycoplasma ovipneumoniae	17.8393	31.2896	0.363	7.899	25.3
Pasteurella multocida	10.2528	19.0445	0.350	7.649	26.1
ORF virus	1.4312	4.0518	0.261	5.960	33.6
Parainfluenza 3 virus (PIV3)	2.1567	8.8356	0.196	4.728	42.3

Notes: ICC(1) = intraclass correlation coefficient representing the proportion of total variance attributable to between-herd differences; design effect = $1 + (m - 1) \times ICC$, where $m = 20$ lambs per herd; effective sample size = $n/$design effect, where $n = 200$ lambs.
