# Supplementary material for: Stage-Specific Associations Between Respiratory Pathogens and Mortality in Sheep: A Multivariate Analysis
Source: Animals (Basel). 2026 Sep 26;16(19):3028. doi: 10.3390/ani16193028 (PMC13663387; doi:10.3390/ani16193028)
Supplement: Supplementary file 1 [file animals-16-03028-s001.zip › Table S2.pdf]

**Table S2.** Distribution of pathogen combinations detected in bronchoalveolar lavage (BAL) samples from 200 lambs. Values are expressed as the number of positive samples and percentage of the total study population. Each animal was assigned to a single mutually exclusive category according to the exact combination of pathogens detected by real-time PCR. Animals in which no pathogen was detected are included as "No pathogen detected".

| Species identified in BAL* | Nº samples (%) |
|---|---|
| *M. haemolytica* + *M. ovipneumoniae* | **54 (27.0%)** |
| *M. haemolytica* | **27 (13.5%)** |
| *M. haemolytica* + *M. ovipneumoniae* + *P. multocida* | **20 (10.0%)** |
| *M. haemolytica* + *B. trehalosi* | **14 (7.0%)** |
| *M. haemolytica* + *M. ovipneumoniae* + *B. trehalosi* | **13 (6.5%)** |
| *M. haemolytica* + *M. ovipneumoniae* + *P. multocida* + *B. trehalosi* | **12 (6.0%)** |
| *M. haemolytica* + *M. ovipneumoniae* + *P. multocida* + PIV3 | **5 (2.5%)** |
| *M. haemolytica* + *M. ovipneumoniae* + *P. multocida* + *B. trehalosi* + *H. somni* | **5 (2.5%)** |
| *M. haemolytica* + *M. ovipneumoniae* + *P. multocida* + *B. trehalosi* + PIV3 | **4 (2.0%)** |
| *M. haemolytica* + *M. ovipneumoniae* + ORF virus | **4 (2.0%)** |
| *M. ovipneumoniae* + ORF virus | **4 (2.0%)** |
| *M. haemolytica* + *B. trehalosi* + *M. ovipneumoniae* + *P. multocida* + PIV3 + *H. somni* | **1 (0.5%)** |
| *M. haemolytica* + *M. ovipneumoniae* + *P. multocida* + PIV3 + *H. somni* + ORF virus | **1 (0.5%)** |
| *M. haemolytica* + *M. ovipneumoniae* + *P. multocida* + *H. somni* + ORF virus | **1 (0.5%)** |
| *M. haemolytica* + *M. ovipneumoniae* + *P. multocida* + PIV3 + ORF virus | **2 (1.0%)** |
| *M. haemolytica* + *M. ovipneumoniae* + *P. multocida* + PIV3 + *H. somni* | **2 (1.0%)** |
| *M. haemolytica* + *M. ovipneumoniae* + *B. trehalosi* + *H. somni* | **1 (0.5%)** |
| *M. haemolytica* + *M. ovipneumoniae* + *B. trehalosi* + ORF virus | **1 (0.5%)** |
| *M. haemolytica* + *M. ovipneumoniae* + PIV3 + *H. somni* | **1 (0.5%)** |
| *M. haemolytica* + *B. trehalosi* + PIV3 | **1 (0.5%)** |
| *M. haemolytica* + *B. trehalosi* + ORF virus | **1 (0.5%)** |
| *M. haemolytica* + *B. trehalosi* + *H. somni* | **2 (1.0%)** |
| *M. haemolytica* + *M. ovipneumoniae* + *P. multocida* + ORF virus | **1 (0.5%)** |
| *M. haemolytica* + *P. multocida* + PIV3 | **1 (0.5%)** |
| *M. haemolytica* + ORF virus | **1 (0.5%)** |
| *M. haemolytica* + *P. multocida* | **1 (0.5%)** |
| *M. haemolytica* + *M. ovipneumoniae* + *PIV3* | **1 (0.5%)** |
| *M. haemolytica* + *M. ovipneumoniae* + *H. somni* | **1 (0.5%)** |
| *M. ovipneumoniae* + *B. trehalosi* | **3 (1.5%)** |
| *M. ovipneumoniae* + PIV3 + ORF virus | **2 (1.0%)** |
| *M. ovipneumoniae* + *B. trehalosi* + ORF virus | **1 (0.5%)** |
| *M. ovipneumoniae* | **2 (1.0%)** |
| *B. trehalosi* | **2 (1.0%)** |
| ORF virus | **1 (0.5%)** |
| No pathogen detected | **2 (1.0%)** |
| TOTAL | **200 (100.0%)** |
