# Supplementary material for: Stage-Specific Associations Between Respiratory Pathogens and Mortality in Sheep: A Multivariate Analysis
Source: Animals (Basel). 2026 Sep 26;16(19):3028. doi: 10.3390/ani16193028 (PMC13663387; doi:10.3390/ani16193028)
Supplement: Supplementary file 1 [file animals-16-03028-s001.zip › Table S3.pdf]

Table S3. Summary of the statistical measures used to evaluate the canonical structure, including their relationship with the original input variables, analytical meaning, interpretation, practical significance, and location within the manuscript.

Measure	Relationship / Variables involved	Works directly with input variables?	What it tells you	Main interpretation/use	Practical significance	Element in which they are shown in Paper
**Standardized canonical coefficients**	Input variable → canonical variate (Y1, Y2)	**Yes**	Relative weight of each input variable in constructing the canonical variate	Describe the **composition** of each canonical function	Larger absolute coefficients suggest greater weighting, but interpretation can be affected by multicollinearity	In text, Section 3.1.3.3. Equations.
**Canonical loadings** (structure coefficients)	Input variable ↔ its canonical variate (Y1, Y2)	**Yes**	Strength and direction of association between each input variable and the canonical variate	Identify which input variables **characterize and define** each canonical function	**	In text, Section 3.1.3.3. Biplot (Figure 3).
**Pearson's Product–Moment Correlations**	Input variable ↔ input variable	**Yes**	Pairwise strength and direction of relationships among the original variables	Examine relationships among predictors and assess **redundancy/multicollinearity**	<0.30 weak; 0.30–0.49 moderate; ≥0.50 strong	Heatmap Chart (Figure 1), Section 3.1.3.1.
**Canonical correlations**	Canonical variate from Set 1 ↔ canonical variate from Set 2	**No**	Strength of the overall relationship between paired canonical dimensions	Assess the **overall association between the two variable sets**	**	In text, Section 3.1.3.3.
**Squared canonical correlation (R^2c)**	Canonical variate ↔ canonical variate	**No**	Proportion of shared variance between paired canonical variates	Quantify the amount of variance shared between the two canonical dimensions	**$R^2c = r^2$**; ≥0.09 corresponds to a canonical correlation of 0.30	In text, Section 3.1.3.3. can be inferred from canonical correlations.

Measure	Relationship / Variables involved	Works directly with input variables?	What it tells you	Main interpretation/use	Practical significance	Element in which they are shown in Paper
**Redundancy coefficient**	Canonical variates → opposite variable set	**Indirectly**	Proportion of variance in one variable set explained by the canonical variate from the other set	Evaluate the **explanatory value** of each canonical function	Higher values indicate greater practical explanatory relevance	In text, Section 3.1.3.3.
**Statistical significance**	Canonical function / canonical correlation	**No**	Whether the observed canonical relationship is statistically distinguishable from zero	Determine which canonical functions are statistically supported	Based on *P*-value or permutation test	Across Manuscript, Result Section
**Practical significance**	Effect magnitude	**Yes / No**	Whether the magnitude of an association is sufficiently large to be substantively relevant	Prevent overinterpretation of statistically significant but weak effects	Based primarily on **effect size/magnitude**, not *P*-value alone	Across Manuscript, Discussion
